# Structure and Ligands Interactions of *Citrus* Tryptophan Decarboxylase by Molecular Modeling and Docking Simulations

**DOI:** 10.3390/biom9030117

**Published:** 2019-03-26

**Authors:** Angelo Facchiano, Domenico Pignone, Luigi Servillo, Domenico Castaldo, Luigi De Masi

**Affiliations:** 1 Consiglio Nazionale delle Ricerche (CNR), Istituto di Scienze dell’Alimentazione (ISA), 83100 Avellino, Italy; 2 CNR, Istituto di Bioscienze e BioRisorse (IBBR), 70126 Bari, Italy; domenico.pignone@ibbr.cnr.it; 3 Dipartimento di Medicina di Precisione, Università degli Studi della Campania “Luigi Vanvitelli”, 80138 Napoli, Italy; luigi.servillo@unicampania.it; 4 Stazione Sperimentale per le Industrie delle Essenze e dei Derivati dagli Agrumi (SSEA), Azienda Speciale della Camera di Commercio di Reggio Calabria, 89125 Reggio Calabria, Italy; dcastaldo@ssea.it; 5 CNR, IBBR, 80055 Portici, Italy

**Keywords:** *Citrus* spp., aromatic l-amino acid decarboxylases, tryptophan decarboxylase, function prediction, modeling, docking

## Abstract

In a previous work, we in silico annotated protein sequences of *Citrus* genus plants as putative tryptophan decarboxylase (pTDC). Here, we investigated the structural properties of *Citrus* pTDCs by using the TDC sequence of *Catharanthus roseus* as an experimentally annotated reference to carry out comparative modeling and substrate docking analyses. The functional annotation as TDC was verified by combining 3D molecular modeling and docking simulations, evidencing the peculiarities and the structural similarities with *C. roseus* TDC. Docking with l-tryptophan as a ligand showed specificity of pTDC for this substrate. These combined results confirm our previous in silico annotation of the examined protein sequences of *Citrus* as TDC and provide support for TDC activity in this plant genus.

## 1. Introduction

Plant natural defense mechanisms attract considerable interest of low-input and sustainable agriculture for crops protection [1]. Aromatic l-amino acids (AA) are precursors of secondary metabolites with defensive roles [2]. The decarboxylation of l-tryptophan and l-tyrosine by specific decarboxylases, dependent on pyridoxal-5′-phosphate (PLP) as a cofactor (vitamin B_6_ derivative), is essential for the production of the respective aromatic amines tryptamine and tyramine [3]. In plants, AA decarboxylases (AADC) evolved significant differences in their substrate selectivity, distinguishing between indole and phenol side chains. Plant l-tryptophan decarboxylase (TDC) is a highly specific lyase (EC 4.1.1.28, formerly EC 4.1.1.27) that exclusively catalyzes the decarboxylation of l-tryptophan and l-5-OH-tryptophan [4]. In fact, TDC does not recognize as substrates phenolic l-amino acids and their derivatives, which instead are specifically recognized by the plant l-tyrosine decarboxylase (TYDC) [5]. Animal AADC admits a broad spectrum of substrates, such as l-5-OH-tryptophan and 3,4-dihydroxy-l-phenylalanine (l-DOPA, used in Parkinson’s disease treatment), and is better known as l-DOPA decarboxylase (DDC) [6].

Both the TDC gene and the enzyme have been characterized in the rosy periwinkle of Madagascar, *Catharanthus roseus* (L.) G. Don, where TDC is composed of two identical subunits (homodimer) encoded by a single-copy gene without intron sequences [4,7,8]. In this medicinal plant, tryptamine is a building block for the monoterpenoid indole alkaloids, including natural anticancer agents, such as vinblastine [3]. Various plants, animals, and fungi contain N-methylated and/or 5-hydroxylated forms of tryptamine that are called monoamine alkaloids o indole alkylamines and are commonly known as “tryptamines” [9]. Several tryptamines are known to induce alteration of consciousness in humans, acting as neurotransmitters or neuromodulators of synaptic transmission [9,10]. Tryptamines were also identified in food plants, such as *Citrus* genus species [11,12]. Our more recent findings showed TDC activity in *Citrus* spp. and in silico identified the candidate protein sequences of TDC in *Citrus* genome [2]. Since alterations of the DDC activity can be associated to neurodegenerative and hypertensive diseases, human and animal DDC have been studied in depth and characterized also from the structural point of view [6,13,14]. On the basis of sequence and secondary structure similarities between animal and plant AADC, an analogous catalytic mechanism was proposed [3,7,13].

Alexander et al. [14] and Sandmeier et al. [15] proposed four groups of amino acid decarboxylases, on the basis of sequence comparison, including AADC into group II. The classification of PLP enzymes evidenced a narrow structural diversity [16]. The PLP enzyme superfamily was subdivided by similarity of primary and secondary structures into five main fold types. Schneider et al. [17] updated and confirmed the structural classification into five PLP-binding folds. Decarboxylases were included into two fold types: group I, II, and III decarboxylases share the PLP-binding motif of fold-type I; fold-type III includes group IV decarboxylases. AADC belong to the group II of fold-type I of aspartate aminotransferase family (alpha family). Efforts to elucidate the structure, precise catalytic mechanism, and substrate specificity of plant AADC are in progress, but the origins of the highly selective substrate specificity of TDC are still unknown [2,3,13].

Plant TDC are challenging to identify through amino acid sequences comparison because of their subtle sequence divergence from AADC [13]. However, because the function of TDC is closely related to its extreme substrate selectivity, differentiation on the base of sequence could be an important advance. The difference in substrate specificity within plant AADC is the result of little, but significant, amino acid variations [2,3,18,19]. Recently, TDC activity was converted to TYDC and vice versa by a single amino acid substitution into the active site [13].

Despite the fact that experimental research is needed to obtain highly accurate functional assignments for metabolic functions and substrate specificity, protein annotations can be sufficiently rigorous if based on in silico data mining and verification of their accuracy. With the aim of identifying structural signatures to confirm the previously proposed in silico functional annotation as TDC of the *Citrus* candidate sequences [2], the present study produced molecular models and substrate docking simulations of the *Citrus* candidate TDC by a computational biology approach, starting from the 3D models of DDC available in human and animals. Analysis of the models for the properties of substrate and PLP binding sites identified the amino acids involved in ligand interaction. The results are in agreement with experimental evidence described in the literature related to the role of given amino acids in homologous sequences, providing novel insights into the structure–function relationships of TDC enzymes.

## 2. Materials and Methods

### 2.1. Comparative Protein Structure Modeling of C. roseus TDC and Citrus pTDCs

The TDC enzyme sequence of *C. roseus* (UniProtKB/Swiss-Prot accession no. P17770) was retrieved from the NCBI database (www.ncbi.nlm.nih.gov) and consists of 500 amino acids deduced in silico from nt 70 to nt 1572 of the 1740 nt coding sequence (GenBank accession no. M25151 and J04521) [7]. The putative TDC (pTDC) sequences of clementine (*Citrus clementina*) cultivar Clemenules and sweet orange (*Citrus sinensis*) cultivar Ridge Pineapple consist of 499 amino acids when translated in silico; they were retrieved from the NCBI database with GenBank accession no. ESR60648 (CICLE_v10014992mg) and KDO55801 (CISIN_1g010842mg), respectively (Ciclev10014992m and orange1.1g010842m in De Masi et al. [2]).

Molecular modeling of the TDCs from *C. roseus* and *Citrus* species sequences identified by a previous research [2], was performed according to the procedures reported in previous papers [20,21]. In brief, comparative (homology) modeling was applied on the basis of the crystallographic structure templates of human DDC [6]. This protein is available in the UniProt database (www.uniprot.org) with sequence accession no. P20711 and in the Protein Data Bank (PDB, www.rcsb.org) with three structures, i.e., 3RBF in the apo form with PLP not covalently bound to A chain, 3RBL in the apo form without PLP, and 3RCH in the open conformation with L-peptide linking (LLP) and PLP bound to the A and B chains, respectively. Among the amino acid sequences of DDCs whose structures are available in PDB, human DDC proved to be the most similar to *C. roseus* TDC and selected *Citrus* pTDC sequences, as obtained with BLAST search. Their alignment showed also similarity levels suitable for applying comparative modeling, having more than 40% sequence identity and nearly complete sequence coverage. The structure of 3RBF, in the apo form with PLP not covalently bound, was used to model the monomers of TDC proteins. In addition, models of the interaction between *Citrus* pTDCs with carbiDOPA inhibitor were similarly created by using the template structure of *Sus scrofa* DDC [18], for which the 1JS3 and 1JS6 structures with and without the inhibitor are available in PDB. Although a little lower than for human DDC, sequence identity and alignment coverage with plant TDCs make this protein also suitable for applying comparative modeling procedures.

Multiple sequence alignments were created by means of the MView tool [22]. Then, the derived alignment of template and target sequences was used as input for MODELLER 9.10 available at www.salilab.org [23]. With the aim of selecting the most reliable model for each *Citrus* pTDC, we generated 10 structural models for each sequence on the basis of stereochemical and energetic data quality, checked by means of PROCHECK [24] and PROSA [25] programs, respectively. The final models were examined with DiscoveryStudio (Dassault Systèmes, Vélizy-Villacoublay, France).

### 2.2. Ligands Docking of Citrus pTDC

Docking simulations for the substrate Trp with the best 3D model of each *Citrus* pTDC, obtained in the previous step, were performed with AutoDock4.0 [26] in order to evaluate the ligand interaction with the enzyme binding site. The ligand was docked in the proposed binding pocket [6], and the software searched for the best interactions between the possible conformations of the substrate and the 3D regions of interest. LigPlot+ [27] was used for investigating in detail the interaction region and creating schematic images of the binding site. Moreover, LigPlot+ was used to analyze the interaction of the carbiDOPA–PLP adduct with sweet orange and clementine pTDC, as modeled on the basis of the PDB template 1JS3 crystallographic structure of *S. scrofa* DDC (see paragraph 2.1).

## 3. Results and discussion

### 3.1. Structure Prediction of TDCs from C. roseus and Citrus by Comparative Modeling

Since animal DDC have shown high sequence similarity to plant TDC [7], we used the crystallographically solved structures of human DDC as a template to model the unknown 3D structure of *C. roseus* TDC monomer by comparative protein modeling (Supplementary Figure S1). The protein of *C. roseus* is annotated as TDC regarding its substrate specificity [7], so its model was our reference to be used for comparison with *Citrus* pTDC which has no experimental annotation.

**Figure 1:**
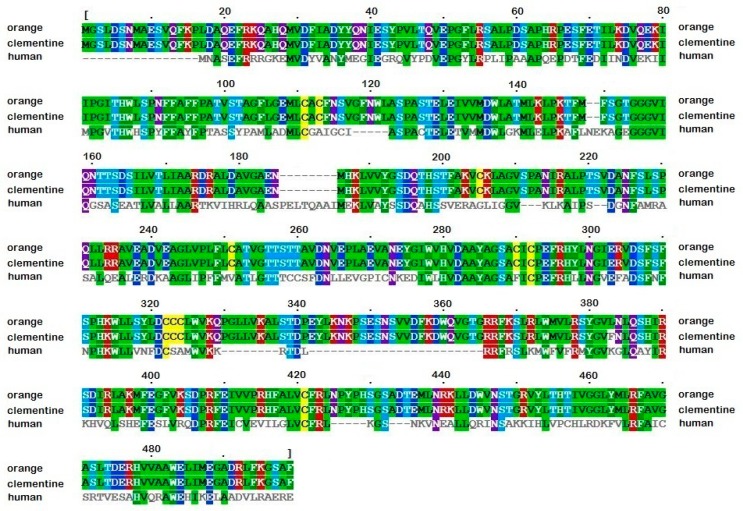
Alignment of sweet orange and clementine putative tryptophan decarboxylases (pTDCs) with human l-DOPA decarboxylase (DDC), used as a template structure for the comparative modeling procedure. The image was adapted by the MView tool [22]. The background color indicates conserved residues, the color code indicates side-chain properties, i.e., green: hydrophobic, cyan: polar, yellow: cysteine, red: positive, blue: negative.

**Figure 2:**
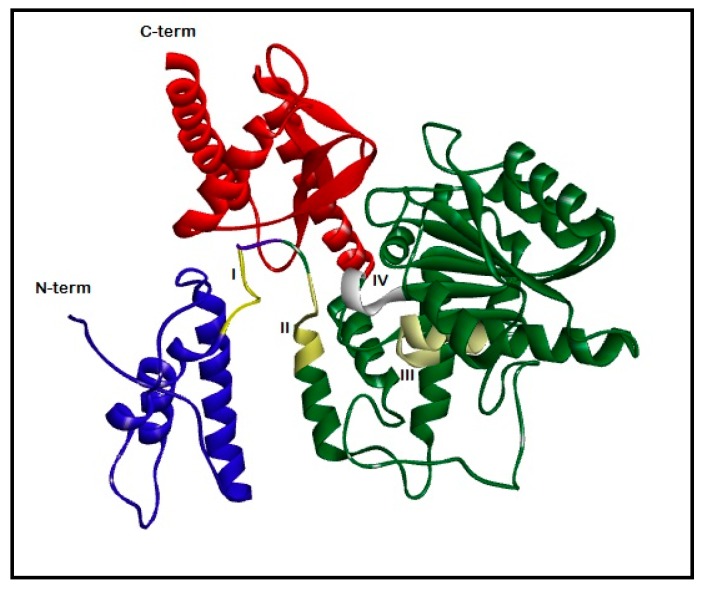
Model of the pTDC subunit from sweet orange. Protein architecture is evidenced by backbone schematization as a ribbon, larger for secondary-structure elements as helices and strands. The three domains are also indicated by colors: blue, N-terminal domain; green, central domain; red, C-terminal domain. The positions of the signature regions are also evidenced: motif I, yellow; II, light yellow; III, very light yellow; IV, white.

Then, we performed a multiple sequence alignment of *Citrus* pTDCs with human DDC (Figure 1) and applied the same procedure of comparative modeling. The two *Citrus* pTDC subunit sequences are different in one amino acid residue (position 383), evidencing close evolutionary relationships. Schematic views of the obtained 3D models of sweet orange and clementine pTDCs are shown in Figure 2 and Supplementary Figure S2, respectively.

Checking the models for their quality, we obtained energy profiles and *Z*-score similar to or better than those of the template structure (see Supplementary Figures S3–S6 and Table S1). Comparative modeling is based on the assumptions that structural similarity matches to functional similarity and that folding of homologous proteins is better conserved than their amino acid sequences; thus, this has been a successful approach [28].

Models of *C. roseus* TDC and *Citrus* pTDCs appear suitable to structural classification in the CATH-Gene3D database [29] for pig and human DDCs [6,18]. The whole architecture of each *Citrus* pTDC subunit consists of three main domains (Figure 2 and Supplementary Figure S2). The first is classified in CATH as 1.20.1340.10 (DOPA decarboxylase, N-terminal domain). It shows the presence of three α-helices, with the up-down bundle architecture of the main alpha structural class. The central domain of the sequence is classified in CATH as 3.40.640.10 (Type I PLP-dependent aspartate aminotransferase-like major domain). It folds as a β-sheet formed by seven strands (six parallel and one antiparallel) surrounded by five α-helices, with α-β sandwich architecture. The last domain is classified in CATH as 3.90.1150.10 (aspartate aminotransferase, domain 1) and consists of a smaller β-sheet, formed by four antiparallel strands, alternated by three α-helices. Few additional helices and short strands are also visible along the whole structure. The association of these secondary structures, known as α/β fold, is similar to what observed in human and animal DDCs [6,18,19].

The models result in agreement with the structural properties expected for TDCs, so we used them for simulations of docking with Trp and PLP in order to gain more information and obtain confirmation of the binding site properties and amino acids involved.

### 3.2. Ligand Binding to TDCs from C. roseus and Citrus by Molecular Docking

**Figure 3:**
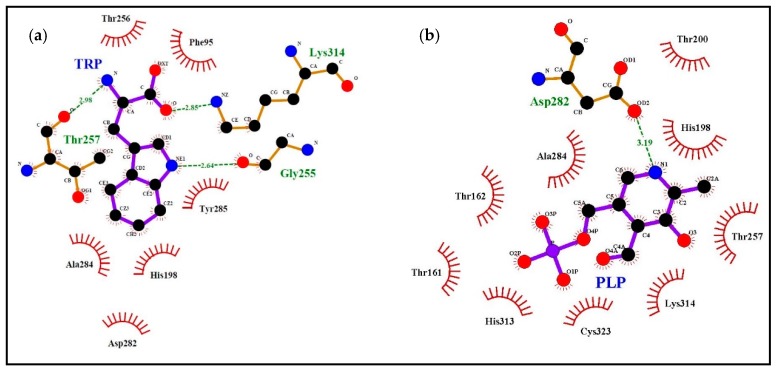
Docking simulations using the model of sweet orange pTDC. The left scheme highlights the interactions with Trp ligand (**a**), the right scheme highlights the interactions with pyridoxal-5′-phosphate (PLP) ligand (**b**). A summary of the interactions is reported in Table 1.

**Figure 4:**
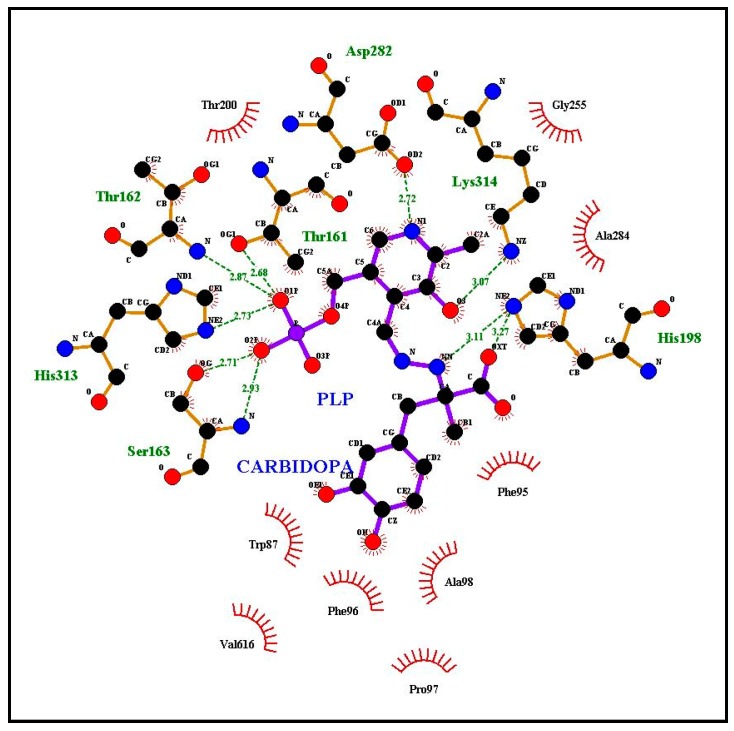
Docking simulation of the carbiDOPA–PLP adduct using the model of sweet orange pTDC. A summary of interactions is reported in Table 1.

We simulated the docking with l-tryptophan and the cofactor PLP of the two *Citrus* pTDCs and of the *C. roseus* TDC model to verify the suitability of binding of the *Citrus* pTDC active site with the substrate Trp (see “Materials and methods” section for details of the procedure). Moreover, because of the availability of a model of pig DDC complexed with carbiDOPA–PLP adduct, we also modeled the sweet orange pTDC with this ligand. A previous TDC comparison, based on amino acid sequence alignments from plant and animal kingdoms, showed high similarity in sequence and structural motifs, demonstrating a remarkable degree of sequence conservation, in spite of the minor substrate selectivity of animal DDC [3,7]. Figure 3 shows the amino acid residues of the sweet orange pTDC binding pocket involved in the interaction with the substrate Trp and the cofactor PLP according to the results of molecular docking simulations. The simulation results for clementine pTDC are shown in Supplementary Figure S7. The analysis of *C. roseus* TDC binding to l-tryptophan was also performed (Supplementary Figure S8), showing the same amino acid residues as those of *Citrus* pTDC interacting with Trp in the docking simulations in Figure 3 and Supplementary Figure S7. The 3D views of the binding pocket and ligands in sweet orange and clementine pTDCs are shown in Supplementary Figures S9 and S10, respectively. We also superimposed the model of sweet orange pTDC docked to Trp on the human DDC structure to evidence the high similarity of protein backbone trace and PLP orientation between model and template (Supplementary Figure S11). Figure 4 shows the interaction of the carbiDOPA–PLP adduct with sweet orange pTDC.

To compare the corresponding amino acids in the 3D models, we listed in Table 1 the residues participating to the interaction with ligands according to our simulations (Figure 3 and Figure 4, Supplementary Figures S7 and S8). In addition, the corresponding amino acid residues observed in TYDCs are reported in the last column of Table 1 (based on the multiple alignment of Supplementary Figure S5 from De Masi et al. [2]). This comparison may be useful to check what residues, potentially involved in ligand interaction, appear conserved or different between plant TDCs and TYDCs. As a general overview, there are several amino acids involved in the interaction with ligands; in fact, some difference is observed when Trp or carbiDOPA is used as a ligand, due to their different structure and steric hindrance. Several amino acids, demonstrated as interacting with ligands as reported in Table 1, belong to the four signatures we evidenced in a previous work [2].

### 3.3. Citrus pTDC Residues Conserved and Involved in Ligand Binding

The first amino acid W87, as indicated in Table 1, interacts with carbiDOPA–PLP in the sweet orange pTDC model (Figure 4) and is part of a conserved motif in TDCs and TYDC. In fact, the segment T_85_[H/N]W[L/M]SP (motif I) is well conserved in TDC proteins with only few differences, reported in brackets. This small motif seems to be able to discriminate TDC from TYDC. The corresponding sequence T_139_HWQSP appears strongly conserved among all TYDC sequences retrieved (Supplementary Figure S5 in De Masi et al. [2]). Within this motif, the discriminating amino acid is the conserved glutamine (Q142) in TYDC, even if this residue does not appear to be directly involved in the interaction with ligands. Nevertheless, this difference is interesting, because glutamine has a polar side chain, while leucine/methionine of TDC have non-polar side chains, suggesting their affinity for Tyr or Trp, respectively. This motif was evidenced in reference [2], and the docking simulation offered further details about its possible role in substrate specificity.

The three amino acids F96, P97, and A98 are components of the segment F_96_PATVSSAAF (motif II), previously evidenced in TDC, and interact with carbiDOPA–PLP in the sweet orange pTDC model (Figure 4). At the corresponding position of TYDCs, we found a serine residue (S152) instead of A98, as reported by Torrens-Spence et al. [13]. This could account for the difference of the substrate specificity: the side chain of the substrate tyrosine is characterized by a hydroxyl group, and so the presence of a serine (side chain: –CH_2_OH) instead of an alanine (side chain: –CH_3_) into the binding site may be useful for substrate recognition and binding. The amino acid that precedes the motif II is F95, also involved in the interaction with ligands in our simulation of interaction with Trp and carbiDOPA–PLP. This residue is Phe or Tyr in TDCs, while it is always Tyr in position 149 of TYDCs, according to the alignment of sequences we reported in our previous study (see Supplementary Figure S5 in De Masi et al. [2]). The variability in position 95 of TDCs induced us to exclude it from the signature of motif II. A detailed investigation (not shown) of the interaction, schematically shown in Figure 3 and Figure 4, revealed that F95 interacts with ligands by means of its main chain atoms, thus suggesting a role that is not sidechain-specific. In our models, the other amino acids of the signature appeared not to be involved in the interaction with the ligands. Moreover, since this region emerged as FPATVSTAGF in our two *Citrus* sequences, this suggests that only the first half of the motif is relevant as a signature for TDCs.

The third segment previously observed in TDC is [H/Q]_159_[G/N]TTSE[A/S]ILCT (motif III), while in TYDC is Q_219_GT[T/A/S][C/S]EA[V/I]L[C/V][T/V]. Our docking results indicated that the central region T_161_TS in TDCs is involved in the interaction with ligands in the *Citrus* pTDC models (Figure 3 and Figure 4, and Supplementary Figure S7). While the first threonine is conserved also in all TYDCs, the other two amino acids in TYDCs are variable. This observation suggests a possible role of this region in the substrate specificity of TDCs and TYDCs. In this motif, we revealed a difference for glutamate (E) in the signature and aspartate (D) in the *Citrus* sequences, a conservative substitution that preserves the carboxylic group in the side chain.

It is particularly useful to note that a complex network of interactions involving numerous other amino acids was revealed by the docking simulations with Trp, carbiDOPA, and PLP in the *Citrus* pTDC models (Figure 3 and Figure 4, and Supplementary Figure S7). A total of nine amino acid residues are involved in the interaction with ligands: H198, T200, G255, T256, T257, D282, A284, Y285, and C323 (Table 1). These residues are not present in the putative signatures proposed in our previous work [2], being well conserved amino acids in both TDCs and TYDCs. Therefore, they appear not to be implied in substrate specificity, although their conservation during evolution evidences their importance in the interaction with ligands. To further confirm this, we observed that the *C. roseus* TDC docking with Trp (Supplementary Figure S8) showed the same conserved residues of the *Citrus* pTDC, as shown by the docking simulations in Figure 3 and Figure 4 and Supplementary Figures S7, S9, S10. Finally, it is interesting to note that H198 and D282 were already indicated to have a role in the catalytic mechanism as proton donor and in PLP stabilization, respectively [30]; the remaining seven amino acid residues, here identified as interacting with ligands, have not yet been experimentally investigated.

The fourth signature previously observed in TDC is S_311_PHKW (motif IV), corresponding to N_371_AHKW in TYDC. This region is known to be part of the PLP-binding domain [31]. The first two amino acids differ in TDCs and TYDCs, thus suggesting a possible role in substrate specificity, even if these two residues do not appear to be directly implied in the interaction with ligands. On the other hand, the imidazole side chain of histidine 313 (H313) appears to interact with the negatively charged phosphate group of PLP and carbiDOPA–PLP (Figure 3 and Figure 4 and Supplementary Figures S7, S9, S10). In addition to this, a key role is ascribed to lysine in position 314 (K314), which takes part in the interaction involving the positive charge of its ε-amino group and the negative charge of the carboxyl group of the Trp ligand (Figure 3, Supplementary Figures S7–S10). This interaction is not related to the side chain of Trp and, therefore, does not seem to be related to the specificity of substrate (i.e., Trp/Tyr preference). However, the presence of a P (proline) or A (alanine) two positions before this lysine may change significantly the backbone trace and the position of K314, because the structure of proline strongly restricts the allowed backbone conformations (Supplementary Figure S9). It is worth of note that the simulation of TDC interaction with Trp locates this ligand in close proximity of this segment, thus suggesting a role for substrate recognition, although more investigations are needed.

In conclusion, modeling and docking simulations allowed us to refine the definition of the amino acids involved in ligand binding sites and plant TDCs function. These interacting amino acids are remarkable, since part of the AADC family active site, whose microarchitecture provides the location where a specific type of substrate can be accepted. In addition, residues with functional groups in their side chains are of certain interest for their potential catalytic function. Therefore, this work provides important indications on which additional amino acid residues of TDCs have to be submitted to experimental analysis using site-directed mutagenesis to verify their real role in the catalytic mechanism. Further clarifications of the specific residues involved in *Citrus* TDC enzymatic reactions will certainly come from the determination of the enzyme 3D structure by X-ray crystallography. Taken all together, these characterized amino acids permit to hypothesize that the *Citrus* protein sequences studied are strong candidates for being homologous to TDC and functionally annotated for that enzymatic activity.

## Figures and Tables

**Table 1:** Plant TDC amino acids involved in the binding of ligands, according to the analysis of the docking simulations. TYDCs, l-tyrosine decarboxylases.

*Citrus* pTDC Amino Acid	Motif ^1^	Interaction with Trp ^2^	Interaction with PLP ^3^	Interaction with carbiDOPA-PLP ^4^	Corresponding Amino Acid in *Catharanthus roseus* TDC ^5^	Corresponding Amino Acid in Plant TYDCs ^6^
W87	I			Yes	W92	W141
F95		Yes		Yes	F100	Y149
F96	II			Yes	F101	F/Y150
P97	II			Yes	P102	P/A151
A98	II			Yes	A103	S152
T161	III		Yes	Yes	T166	T/S221
T162	III	Yes	Yes	Yes	T167	T/A/G/S222
S163	III			Yes	S168	C/S223
H198		Yes	Yes	Yes	H203	H/L258
T200			Yes	Yes	M205	A/S260
G255		Yes		Yes	G260	G315
T256		Yes			T261	T316
T257		Yes	Yes		T262	T317
D282		Yes	Yes	Yes	D287	D342
A284		Yes	Yes	Yes	A289	A344
Y285		Yes			Y290	Y345
H313	IV		Yes	Yes	H318	H373
K314	IV	Yes	Yes	Yes	K319	K374
C323			Yes		T328	C/S383

^1^ The Latin numbering indicates the four motifs (I, II, III, IV) reported by De Masi et al. [2]. ^2, 3^ Interaction with Trp or PLP for sweet orange and clementine pTDC as graphically described in Figure 3 and Supplementary Figure S7, respectively. ^4^ Interaction with carbiDOPA–PLP for sweet orange pTDC as graphically described in Figure 4. ^5^ The amino acid numbering is according to sequence P17770. ^6^ The amino acid numbering is according to multiple sequence alignment reported in Supplementary Figure S5 of De Masi et al. (2017) [2].

## Supplementary Materials

The following are available online at https://www.mdpi.com/2218-273X/9/3/117/s1. Figure S1: Model of *C. roseus* TDC subunit; Figure S2: Model of pTDC subunit from clementine; Figure S3: Energy profile from PROSA Web of Template X-ray structure – PDB code: 3RBF; Figure S4: Energy profile from PROSA Web of *C. roseus* TDC; Figure S5: Energy profile from PROSA Web of sweet orange pTDC; Figure S6: Energy profile from PROSA Web of clementine pTDC; Figure S7: Docking simulations with the model of clementine pTDC; Figure S8: Docking simulation with the model of *C. roseus* TDC; Figure S9: 3D representation of ligand binding site in sweet orange pTDC; Figure S10: 3D representation of ligand binding site in clementine pTDC; Figure S11: Superposition of modeled sweet orange pTDC and template protein (human DDC, PDB code: 3RBF); Table S1: Quality checks for the models of TDC from *C. roseus* and pTDCs from *Citrus* by means of ProSA-Web.
