# Supplementary material for: Structure and Ligands Interactions of Citrus Tryptophan Decarboxylase by Molecular Modeling and Docking Simulations
Source: Biomolecules. 2019 Mar 26;9(3):117. doi: 10.3390/biom9030117 (PMC6468663; doi:10.3390/biom9030117)
Supplement: Supplementary file 1 [file biomolecules-09-00117-s001.pdf]

SUPPLEMENTARY MATERIALS

Structure and Ligands Interactions of *Citrus* Tryptophan Decarboxylase by Molecular Modeling and Docking Simulations

Angelo Facchiano [1,*], Domenico Pignone [2], Luigi Servillo [3], Domenico Castaldo [4] and Luigi De Masi [5,*]

[1] Consiglio Nazionale delle Ricerche (CNR), Istituto di Scienze dell'Alimentazione (ISA), Avellino, 83100, Italy; angelo.facchiano@isa.cnr.it

[2] CNR, Istituto di Bioscienze e BioRisorse (IBBR), Bari, 70126, Italy; domenico.pignone@ibbr.cnr.it

[3] Dipartimento di Medicina di Precisione, Università degli Studi della Campania "Luigi Vanvitelli", Napoli, 80138, Italy; luigi.servillo@unicampania.it

[4] Stazione Sperimentale per le Industrie delle Essenze e dei Derivati dagli Agrumi (SSEA), Azienda Speciale della Camera di Commercio di Reggio Calabria, Reggio Calabria, 89125, Italy; dcastaldo@ssea.it

[5] CNR, IBBR, Portici, 80055, Italy; luigi.demasi@ibbr.cnr.it

* Correspondence: angelo.facchiano@isa.cnr.it (A.F.); luigi.demasi@ibbr.cnr.it (L.D.M.).

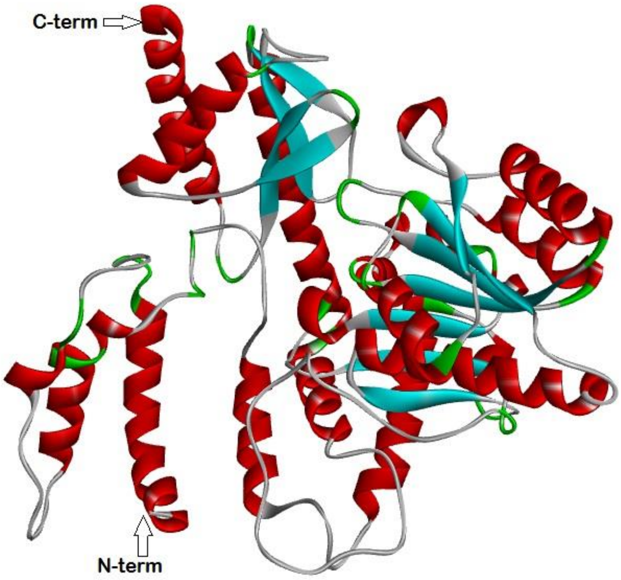

Figure S1.

Model of *C. roseus* TDC subunit.

Protein architecture is evidenced by backbone schematization as a ribbon, larger for secondary structure elements as helices and strands. N-terminal and C-terminal residues are indicated by arrows.

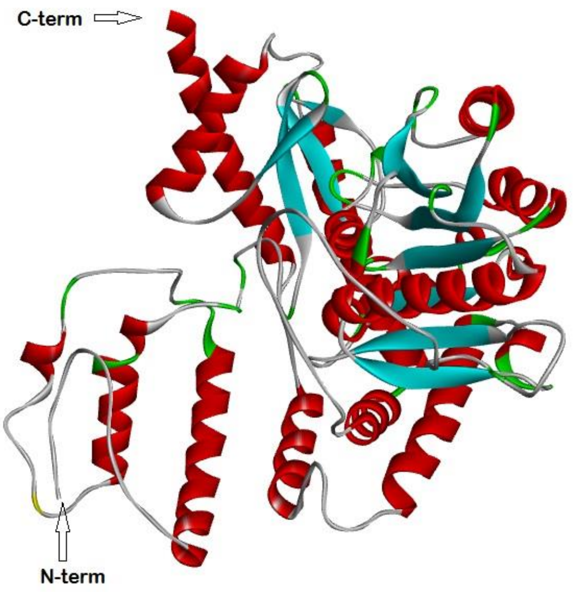

Figure S2.

Model of pTDC subunit from clementine.

Protein architecture is evidenced by backbone schematization as a ribbon, larger for secondary structure elements as helices and strands. N-terminal and C-terminal residues are indicated by arrows. Model shows the same architecture of sweet orange pTDC (see Figure 1) and is similarly oriented to help comparison.

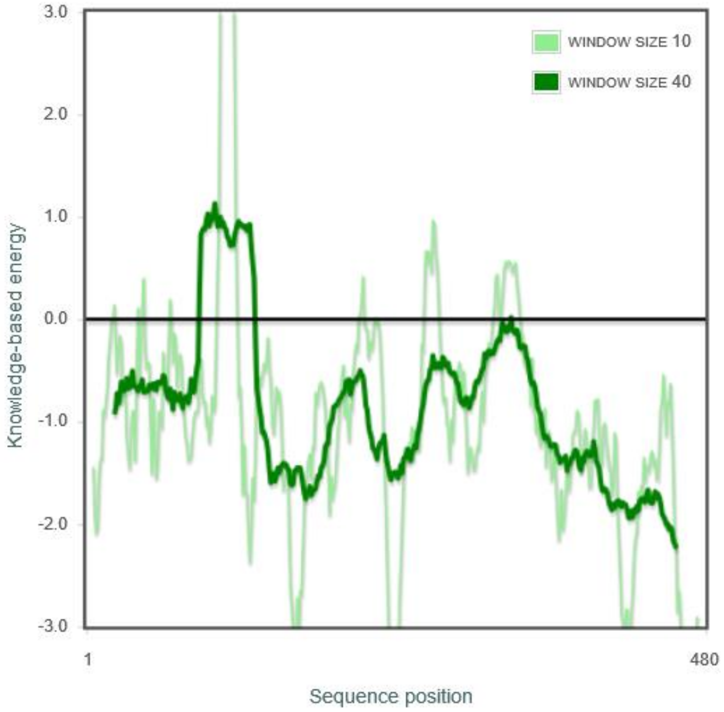

Figure S3.

Energy profile from PROSA Web of Template X-ray structure – PDB code: 3RBF.

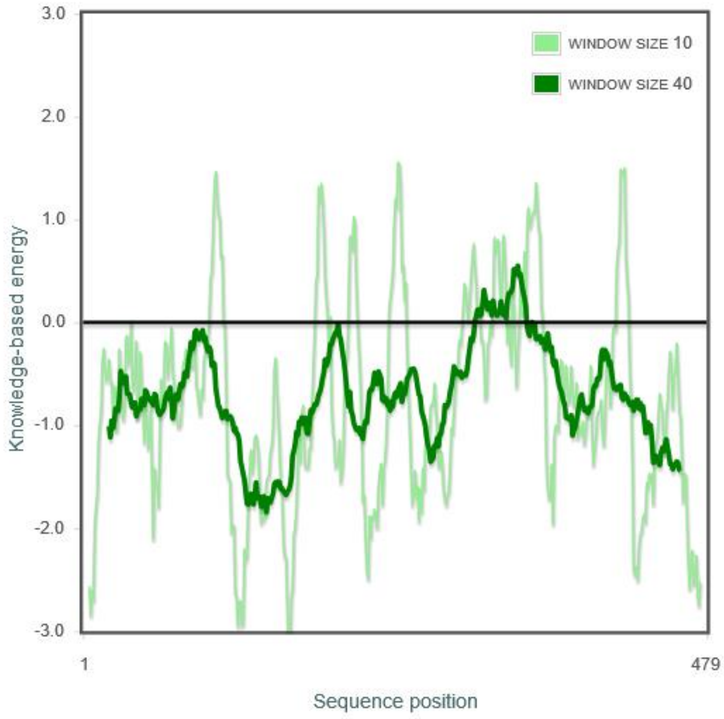

Figure S4.

Energy profile from PROSA Web of *C. roseus* TDC.

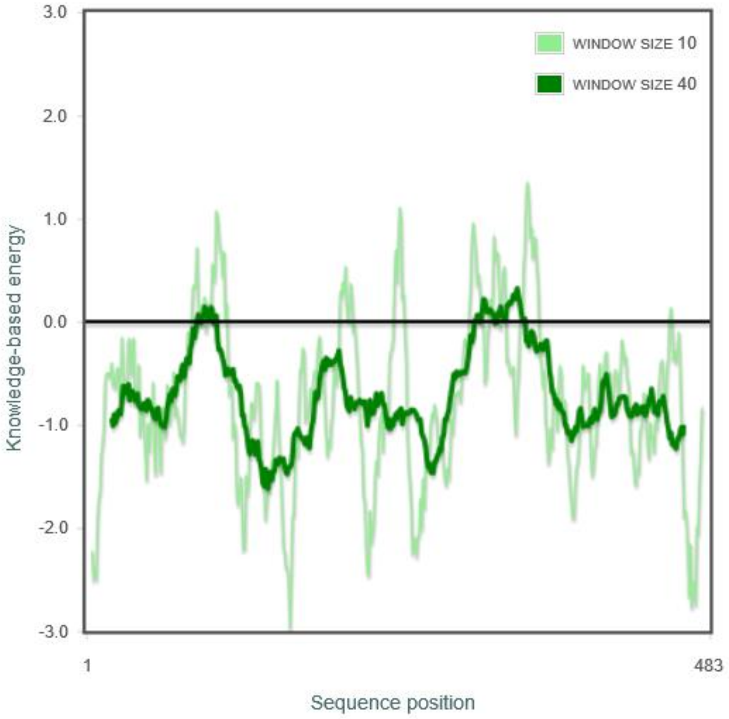

Figure S5.

Energy profile from PROSA Web of sweet orange pTDC.

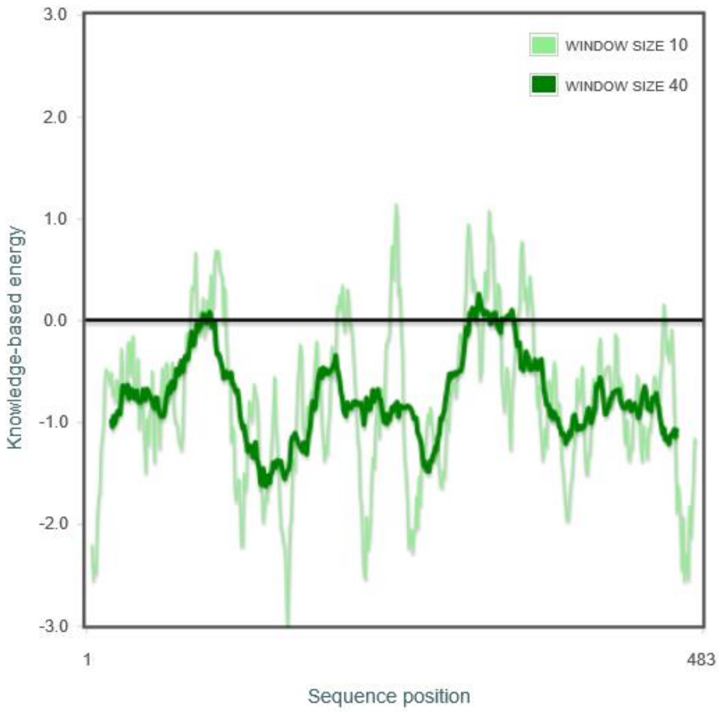

Figure S6.

Energy profile from PROSA Web of clementine pTDC.

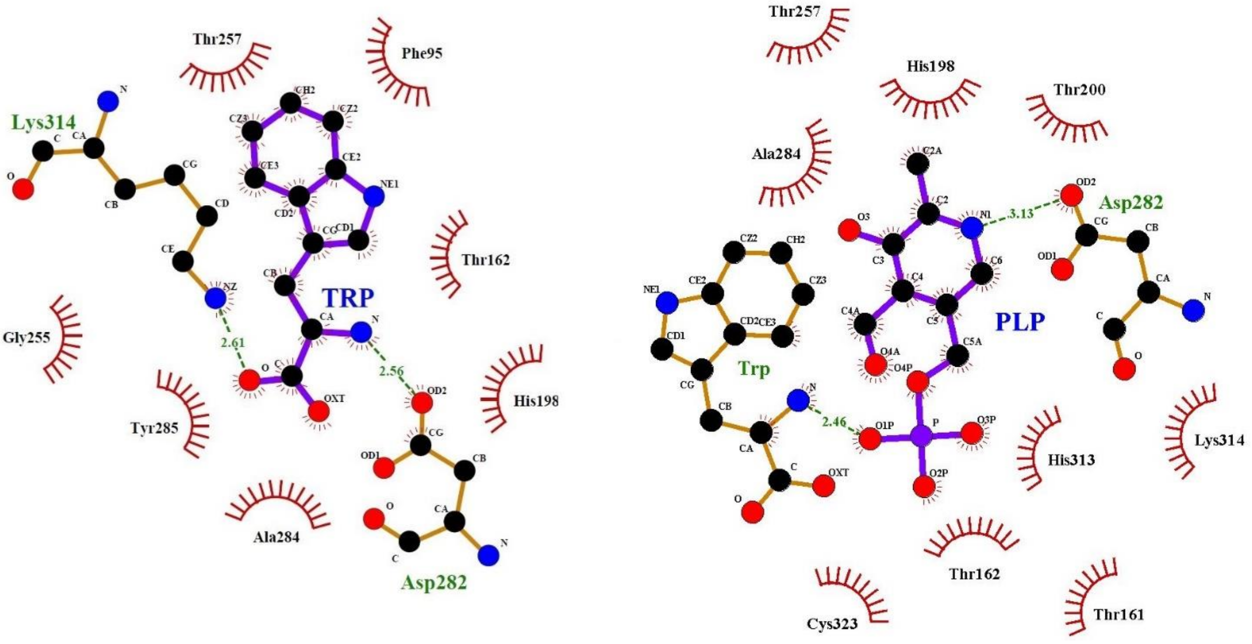

Figure S7.

Docking simulations with the model of clementine pTDC.

The left scheme highlights interactions with Trp ligand, the right scheme highlights interactions with PLP. A summary of the interactions is reported in Table 1.

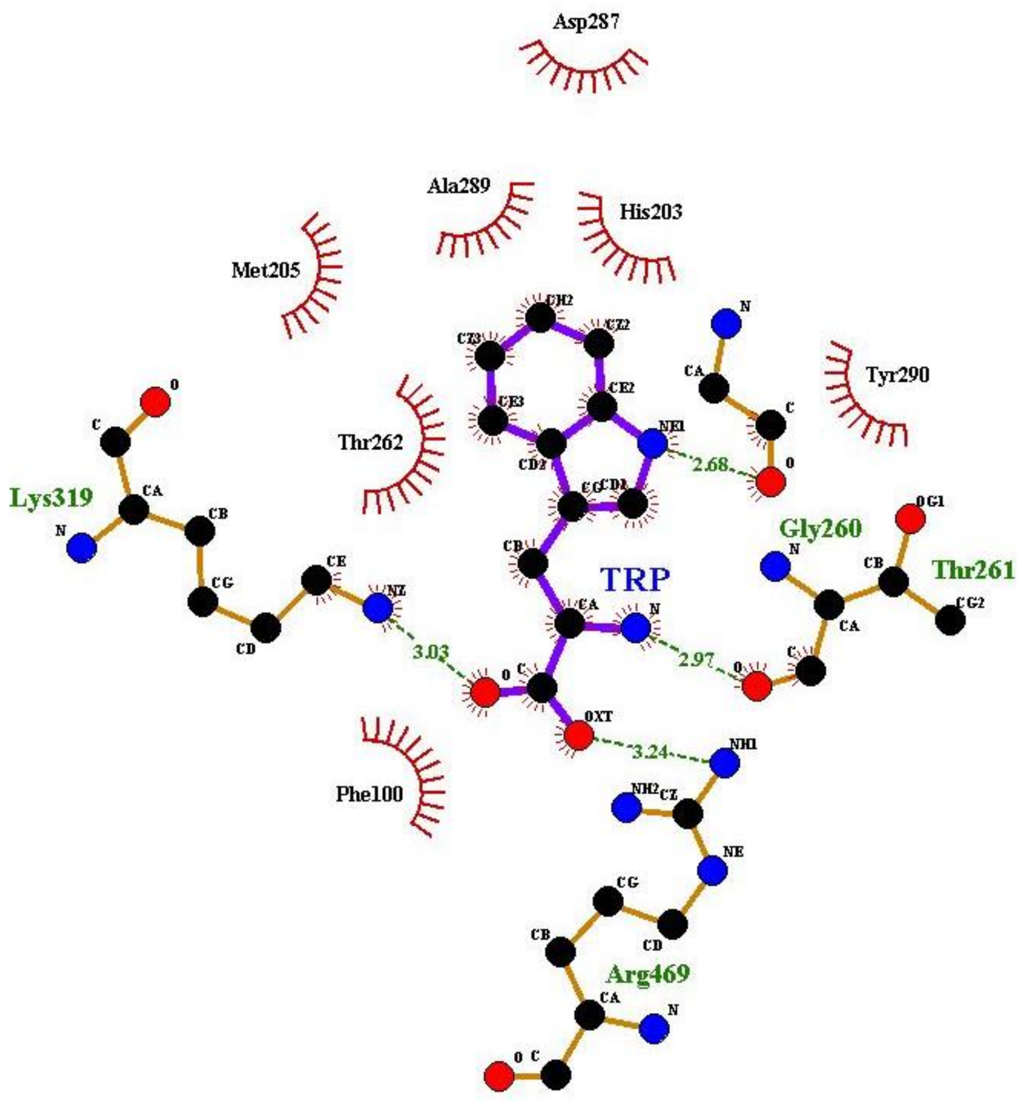

Figure S8.

Docking simulation with the model of *C. roseus* TDC.

The scheme highlights interactions with Trp ligand.

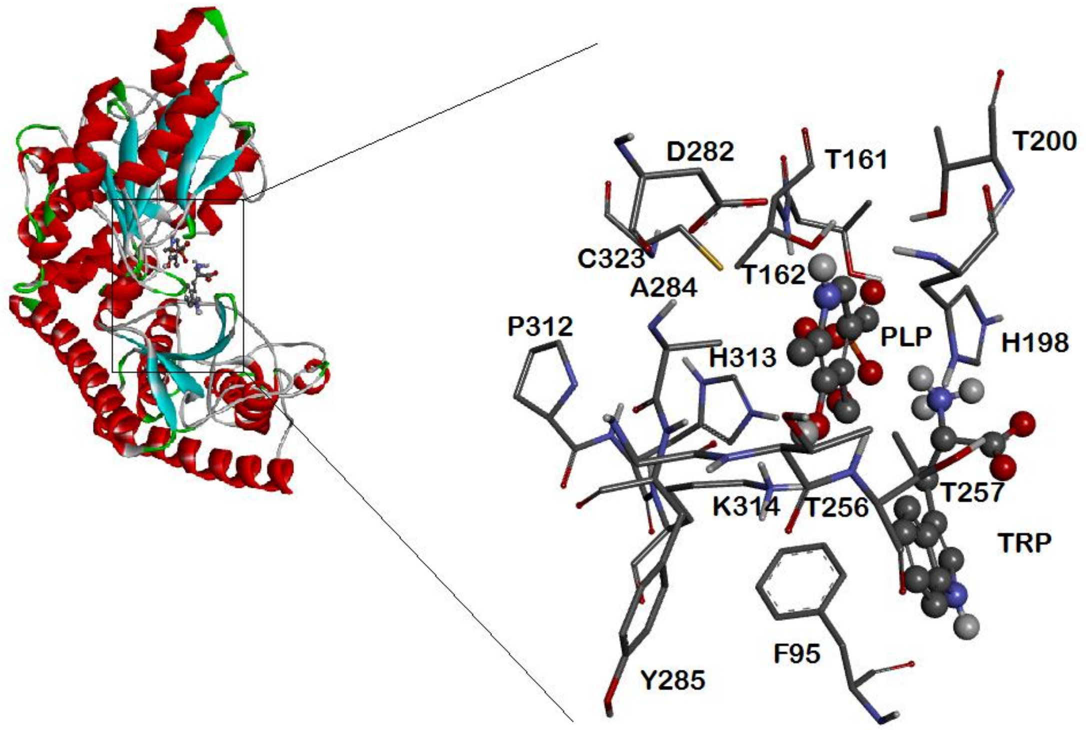

Figure S9.

3D representation of ligand binding site in sweet orange pTDC.

On the left, the whole protein structure is shown. On the right, the detail of the binding site. PLP and Trp ligands are shown in ball-and-stick representation. Amino acids involved in the interaction with PLP and Trp (see Table 1 for the list) are shown in stick representation.

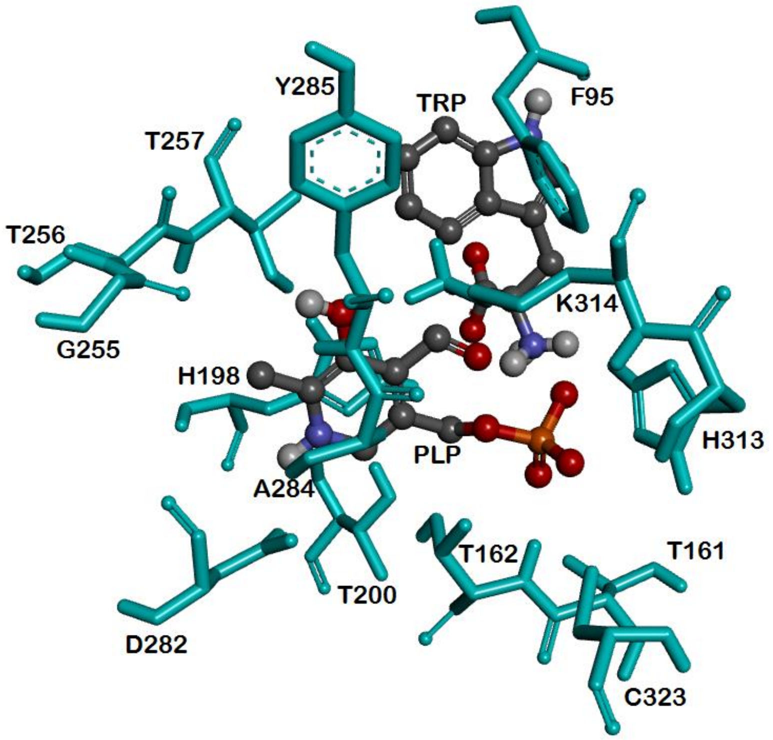

Figure S10.

3D representation of ligand binding site in clementine pTDC.

The detail of the binding site in clementine pTDC, with both PLP and TRP, is shown with an orientation differing from Figure S10 in order to offer a different point of view. PLP and Trp ligands are shown in ball-and-stick representation. Amino acids involved in the interaction with PLP and Trp (see Table 1 for the list) are shown in stick representation.

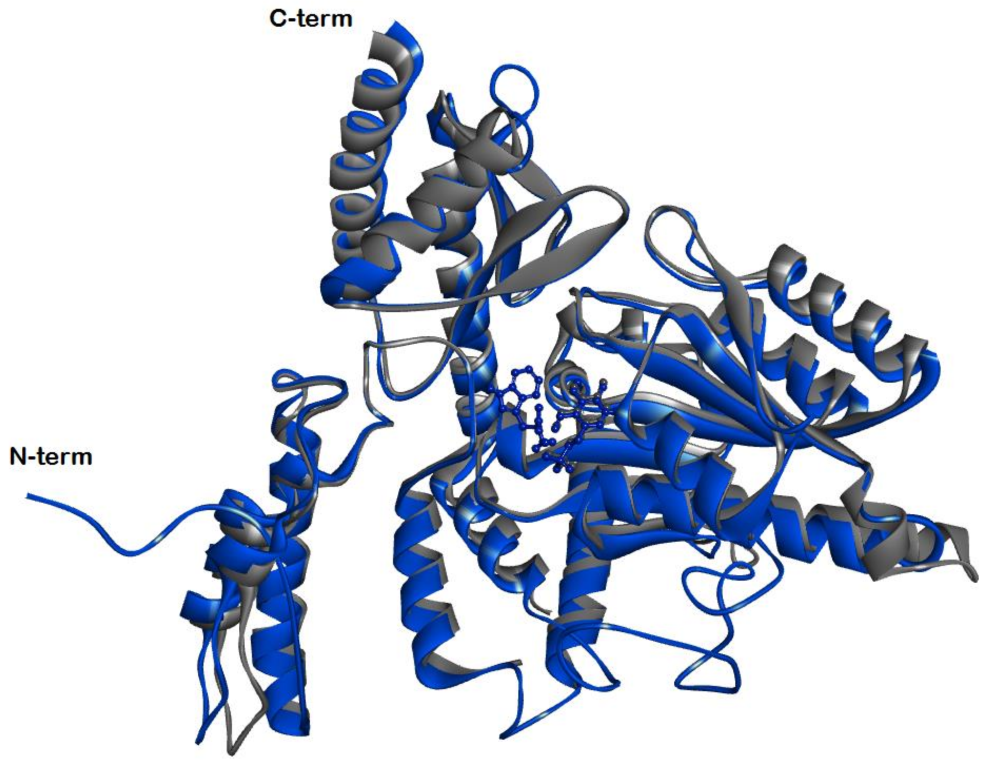

Figure S11.

Superposition of modeled sweet orange pTDC and template protein (human DDC, PDB code: 3RBF).

The model is shown in blue, the template is show in grey. In the center of the figure, ligands are shown in ball-and-stick representation. The PLP molecules from both modeled and template structures are coloured in blue and grey, respectively. Trp is not present in the template structure, so it is shown (in blue) only the molecule docked to the sweet orange pTDC in our simulations.

Table S1. Quality checks for the models of TDC from *C. roseus* and pTDCs from *Citrus* by means of ProSA-Web [1]

**Protein structure**	**Z-score**
Template X-ray structure - PDB code: 3RBF	-9.26
C. roseus TDC subunit	-9.76
Sweet orange pTDC subunit	-9.81
Clementine pTDC subunit	-10.12

[1] References:

- Wiederstein, M.; Sippl, M.J. ProSA-web: interactive web service for the recognition of errors in three-dimensional structures of proteins. *Nucleic Acids Research* **2007**, 35, W407-W410.

- Sippl, M.J. Recognition of Errors in Three-Dimensional Structures of Proteins. *Proteins* **1993**, 17, 355-362.
